# Monitoring Nanoparticle Interaction with Murine Breast Cancer Cells Using Multimodal Fluorescence Lifetime Microscopy

**DOI:** 10.3390/ijms27031339

**Published:** 2026-01-29

**Authors:** Steven Eckstein, Louisa Herbsleb, Henriette Gröger, Claus Feldmann, Frauke Alves, Andreas Walter, Herbert Schneckenburger

**Affiliations:** 1 Center for Optical Technologies, Aalen University, Beethovenstr. 1, 73430 Aalen, Germany; steven.eckstein@studmail.htw-aalen.de (S.E.); louisa.herbsleb@hs-aalen.de (L.H.); andreas.walter@hs-aalen.de (A.W.); 2 Institute of Inorganic Chemistry, Karlsruhe Institute of Technology (KIT), Engesserstr. 15, 76131 Karlsruhe, Germany; henriette.groeger@kit.edu (H.G.); claus.feldmann@kit.edu (C.F.); 3 Translational Molecular Imaging, Max-Planck-Institute for Multidisciplinary Sciences—City Campus, Hermann-Rein Str. 3, 37075 Göttingen, Germany; falves@gwdg.de

**Keywords:** light microscopy, fluorescence lifetimes, inorganic–organic hybrid nanoparticles (IOH-NPs), cellular location, FRET

## Abstract

To investigate drug delivery in cancer therapy, we integrate fluorescence lifetime measurements, microspectrometry, and confocal laser scanning microscopy to track the uptake of inorganic–organic hybrid nanoparticles (IOH-NPs) by breast cancer cells over incubation periods ranging from 2 to 24 h. Non-radiative energy transfer (FRET) from the LysoTracker Green to the IOH-NPs confirms their lysosomal localization and possibly improves their optical excitation. Beyond the resolution limits of light and electron microscopy, fluorescence lifetime kinetics—including FRET—can thus reveal the nanoscale cellular localization of IOH-NPs and guide the optimization of fluorescence excitation. Here, we extend optical microscopy into a fifth dimension—picosecond fluorescence decay times—complementing 3D spatial and spectral information, establishing lifetime measurements as a versatile tool to study nanoparticle uptake in cancer therapy.

## 1. Introduction

**Figure 1:**
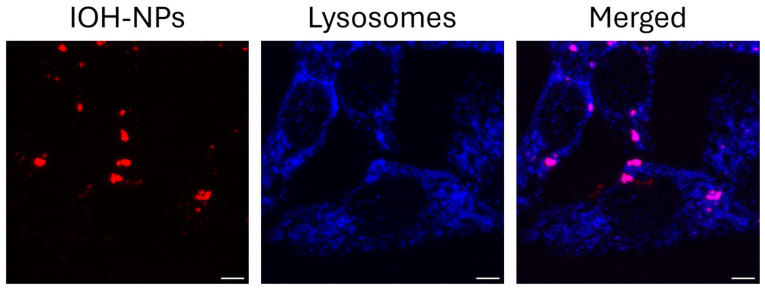
Laser scanning microscopy of H8N8 breast cancer cells incubated for 24 h with IOH-NPs (excitation wavelength: 639 nm, detection range: ≥640 nm, red) and 30 min with LysoTracker Blue (excitation wavelength: 405 nm, detection range: 420–480 nm); scale bar: 5 µm. Reproduced from [5] with modifications.

Inorganic–organic hybrid nanoparticles (IOH-NPs) are promising for drug delivery in nanomedicine, especially in oncology [1,2,3,4]. They can be loaded with chemotherapeutic agents and offer a promising approach for cancer therapy by enabling precise drug delivery, minimizing side effects, improving therapeutic efficacy, and ultimately increasing patient survival [2]. In a correlative light and electron microscopy (CLEM) study, we recently visualized and quantified the uptake and cellular distribution of IOH-NPs in H8N8 murine breast cancer cells [5,6,7]. IOH-NPs were internalized by endocytosis and accumulated in lysosomal vesicles within 24 h, as shown in Figure 1 after co-incubation with LysoTracker Blue.

### 1.1. Inorganic-Organic Hybrid Nanoparticles (IOH-NPs)

IOH-NPs were developed as versatile, high-drug-load nanocarriers (>60% of the nanoparticle mass) for theranostics (therapy + diagnostics). IOH-NPs are characterized by a saline composition consisting of an inorganic cation and a fluorescent dye and/or drug anion. The dye/drug anion is functionalized by phosphate, sulfonate, or carboxylate groups. In combination with a suitable cation, e.g., zirconyl ([ZrO]^2+^), the drug/drug anions form poorly soluble compounds in water [1,2]. For a first study on uptake and cellular localization, all experiments presented here were performed with Dyomics-647 uridine triphosphate (DUT647) fluorescence-labeled reference IOH-NPs without an active chemotherapeutic agent to prevent premature cell death and to visualize the localization of the IOH-NPs without antiproliferative effects. Specifically, [ZrO]^2+^[(CMP)_0.99_(DUT647)_0.01_]^2−^ IOH-NPs (CMP: cytidine monophosphate), 50–60 nm in size, containing a very low amount of DUT647 as an intensely emissive fluorophore, were used.

These IOH-NPs have been previously characterized in detail [3]. The incorporation of DUT647 into the IOH-NPs enables a direct visualization of their intracellular distribution using fluorescence microscopy [2]. In in vitro studies, the intracellular uptake of IOH-NPs was confirmed, and their anti-cancer effect was shown in a murine breast cancer cell line (pH8N8) and a human pancreatic cancer cell line (AsPC1) [3]. In AsPC1 cells, reference IOH-NPs ended up in LysoTracker-positive late endosomes and lysosomes [2], as later confirmed by 3D-CLEM of single pH8N8 cancer cells [5].

### 1.2. Multimodal Fluorescence Lifetime Microscopy

The purpose of the present paper is to complement the 3D CLEM measurements via multimodal optical methods, thus proving the cellular location of IOH-NPs in the context of cell organelle-specific local physicochemical conditions. Information can be retrieved in the nanometer range, i.e., below the optical resolution according to the Abbe criterion Δx ≥ λ/2A_N_ or the Rayleigh criterion Δx = 0.61 λ/A_N_ (with the numerical aperture A_N_). In particular, we combined fluorescence lifetime microscopy (FLIM) with microspectrometry and confocal laser scanning microscopy (C-LSM) to study the uptake and location of IOH-NPs on the basis of their fluorescence lifetimes. In contrast to fluorescence intensities, fluorescence lifetimes are largely independent of fluorophore concentration and the intensity of irradiation. Furthermore, they reflect a sum of radiative and non-radiative transitions from an excited molecular state, with the latter ones depending on molecular environments or intermolecular interactions, such as pH values, polarity, refractive index, or oxygen concentration [8,9,10]. In the present manuscript, we show that fluorescence lifetime measurements support the localization of IOH-NPs within the lysosomes after cellular uptake. In addition, we report on a potential non-radiative energy transfer from the fluorescently labeled lysosomal marker LysoTracker Green to the IOH-NPs.

## 2. Results

### 2.1. Time-Dependent Fluorescence Lifetime and Spectroscopy Experiments

**Figure 2:**
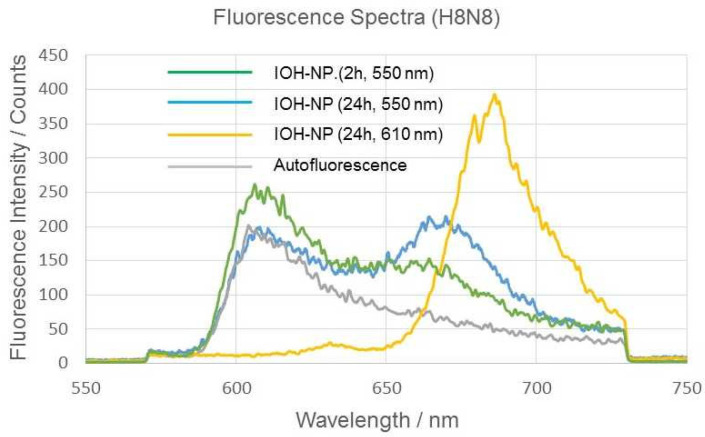
Fluorescence spectra of H8N8 cells incubated with IOH-NPs for 2 h or 24 h compared to non-incubated H8N8 control cells (exhibiting autofluorescence). A total of 5–8 cells were illuminated at (550 ± 20) nm, and fluorescence was detected in a range of 570 nm–730 nm using a 63×/0.90 water immersion microscope objective lens. For comparison, an emission spectrum of H8N8 cells incubated with IOH-NPs for 24 h, excited at (610 ± 10) nm, and detected at λ ≥ 645 nm is added.

**Figure 3:**
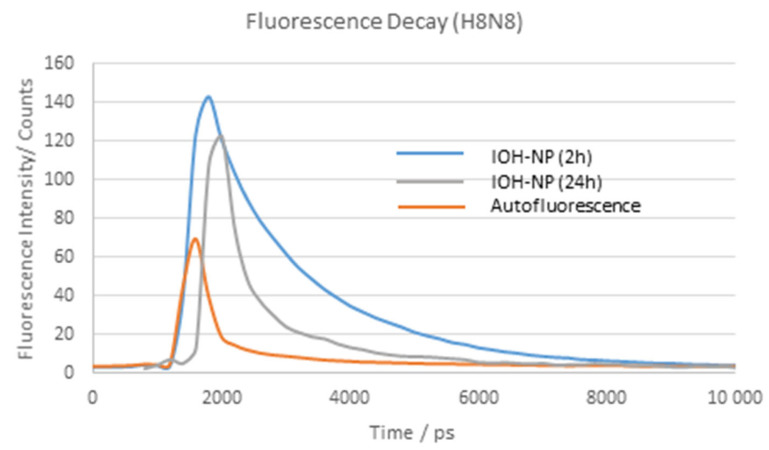
Fluorescence decay kinetics of H8N8 cells incubated with IOH-NPs for 2 h or 24 h compared to non-incubated H8N8 control cells (exhibiting autofluorescence). A total of 5–8 cells were illuminated by picosecond laser pulses at (550 ± 20) nm, and fluorescence was detected in steps of 200 ps over a range of 10 ns at λ ≥ 590 nm using a 63×/0.90 water immersion microscope objective lens.

After incubation of murine breast H8N8 tumor cells with DUT647-labeled reference IOH-NPs for 2 or 24 h, the fluorescence spectra of the cells show an emission band with a maximum around 670–680 nm, which, in the case of 550 nm excitation, is superposed by a broad background of autofluorescence. This background can be minimized when the excitation wavelength λ_ex_ is shifted from 550 nm to 610 nm (see Figure 2). Figure 2 also proves an increase in nanoparticle emission and a shift in the emission maximum from 670 nm to 680 nm when λ_ex_ is shifted from 550 nm to 610 nm (in the latter case, however, some minor part of fluorescence at the short-wave edge of the emission band may be cut by the long pass filter for 645 nm). As the incubation time of the nanoparticles increases, their fluorescence intensity increases but does not show any spectral shift. Fluorescence decay curves are depicted in Figure 3 after incubation of the breast cancer cells with IOH-NPs for 2 or 24 h, together with a reference curve of non-incubated cells. Decay curves were fairly tri-exponential with fluorescence lifetimes of τ_1_ = 200–300 ps, τ_2_ = 1.4–1.7 ns, and τ_3_ in the 100 ns up to the µs range, corresponding to autofluorescence, IOH-NPs, and background fluorescence, respectively. τ_1_ and τ_3_ were also detected in control cells without IOH-NPs. From 33 measurements of 5–10 cells each at an incubation time of 2 h with IOH-NPs, and 26 measurements of 5–10 cells each at an incubation time of 24 h, we calculated median values ± MADs of τ_2_ = (1.70 ± 0.10) ns for 2 h and (1.40 ± 0.24) ns for 24 h incubation. These decay times confirmed earlier measurements of 3T3 murine fibroblasts. They were independent from the excitation wavelength (550 nm or 610 nm) and did not change upon co-incubation of the cells with LysoTracker Green (see Section 2.3). The change in fluorescence lifetimes may be correlated with different microenvironments the IOH-NPs encounter, e.g., mainly outside the cells after 2 h and predominantly within the cells (e.g., within an acidic compartment such as lysosomal vesicles) after 24 h, as described in [5]. In addition, an intrinsic change in fluorescence lifetimes of the IOH-NPs from (1.68 ± 0.08) ns to (1.47 ± 0.14) ns (medians ± MADs of 15 measurements each) in culture medium within 24 h should be considered.

To test more generally whether fluorescence lifetimes of IOH-NPs are shortened in an acidic environment, we measured their decay kinetics at pH7 and pH4. While medians ± MADs of 16 individual lifetime values were almost identical (pH7: (1.80 ± 0.29) ns; pH4: (1.81 ± 0.26) ns), their mean values ± standard deviations differed between (2.667 ± 1.517) ns (pH7) and (1.885 ± 0.466) ns (pH4), thus indicating a shortening with decreasing pH.

### 2.2. Fluorescence Lifetime Images

Effective fluorescence lifetimes τ_eff_, which would result from mono-exponential curve fitting, could also be visualized as fluorescence images. Therefore, fluorescence intensities were detected in two time gates of 0.5 ns width each, which were shifted by 2 ns between one another, and τ_eff_ was calculated, as reported in Section 4.4. For H8N8 breast cancer cells incubated for 24 h with IOH-NPs, effective fluorescence lifetimes between 2.5 ns and 3.5 ns were distributed homogenously over the cells and confirmed cellular uptake of these nanoparticles. Due to an optical resolution around 0.22 µm (according to the Rayleigh criterion) and a pixel resolution of the image intensifying camera system of 0.35 µm, single IOH-NPs with a size of 50–60 nm could not be resolved, and only average values were presented (see Supplementary Figure S1). Effective lifetimes of non-incubated H8N8 (control) cells were about 0.5–1.0 ns and represented small amounts of autofluorescence and scattered light without any discernible cellular structure (due to very low fluorescence intensity).

### 2.3. Influence of the LysoTracker Green on IOH-NPs

**Figure 4:**
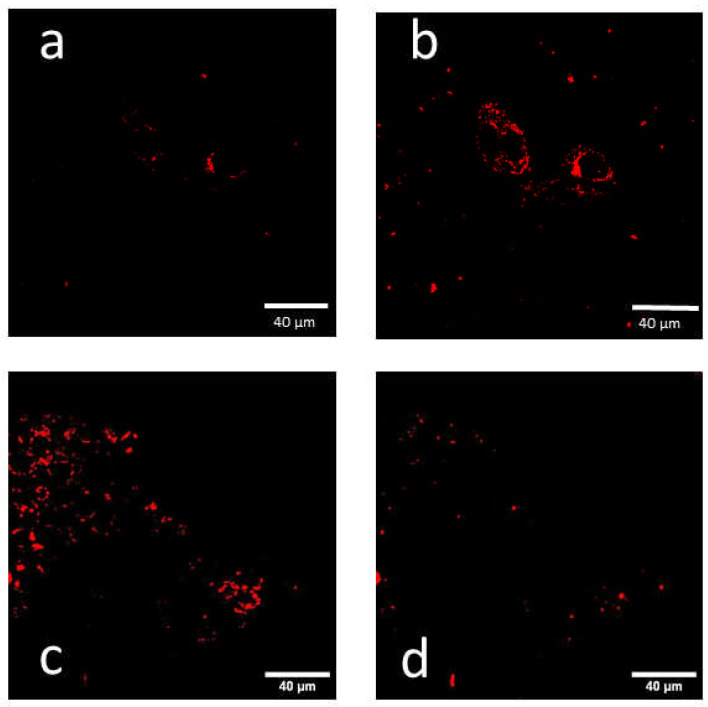
Confocal fluorescence images of H8N8 cells after incubation with IOH-NPs (24 h) (**a**,**b**) and after co-incubation with IOH-NPs (24 h) and LysoTracker Green (30 min) (**c**,**d**) recorded at λ ≥ 650 nm; excitation wavelength: 555 nm (**a**,**c**) or 639 nm (**b**,**d**). Both (**a**,**b**) and (**c**,**d**) image pairs show the same nanoparticles (scale bar: 40 µm). After incubation of H8N8 cells (only) with IOH-NPs, fluorescence excitation is more pronounced at 639 nm, but it is more pronounced at 555 nm after co-incubation with IOH-NPs and LysoTracker Green.

Using C-LSM, we excited IOH-NPs at 555 nm with a laser power of 0.5 mW and 639 nm with a laser power of 0.2 mW. Since the fluorescence excitation of the incorporated dye DUT647 was about 10 times more efficient at λ_ex_ = 639 nm [11], the IOH-NPs (recorded at λ ≥ 650 nm) appeared brighter when excited at this wavelength (Figure 4a,b, see also the fluorescence spectra in Figure 2). After co-incubation of H8N8 cells with IOH-NPs and LysoTracker Green, fluorescence intensities of the IOH-NPs were similar at excitation wavelengths of 555 nm and 639 nm and in a few cases even stronger at λ_ex_ = 555 nm (see Figure 4c,d). The question arises whether the additional fluorescence of the LysoTracker Green, excited at 555 nm, contributes to the fluorescence image or whether the excitation of the IOH-NPs becomes more efficient after optical excitation of the LysoTracker and non-radiative energy transfer (FRET) [12,13,14] to the IOH-NPs. Fluorescence of the LysoTracker Green at an excitation wavelength of 550 nm is well documented by our emission spectra in the range of 600–750 nm (see Table 1). To investigate whether non-radiative FRET from the LysoTracker Green to the IOH-NPs may cause further excitation of the IOH-NPs at 555 nm, we measured the fluorescence lifetimes of the LysoTracker: When excited at 470 nm, the LysoTracker Green showed two fluorescence lifetimes of τ_1_ = 0.5–0.7 ns and τ_2_ slightly above 4 ns. τ_2_ decreased from (4.36 ± 0.20) ns when cells were only incubated with the LysoTracker to (4.05 ± 0.30) ns after co-incubation of the dye with IOH-NPs. These values represent median values ± MADs determined from 11 measurements in the first case and 18 measurements in the second case. After replacement of median values ± MADs by mean values ± standard deviations, τ_2_ decreased from (4.42 ± 0.27) ns after incubation of H8N8 cells with the LysoTracker to (3.97 ± 0.55) ns after co-incubation of the LysoTracker Green and IOH-NPs. According to a Welch t-test (assuming unequal variances) [15], these differences were statistically significant with a level of significance *p* ≤ 0.01. With the fluorescence lifetime corresponding to the inverse rate 1/k = 1/(k_f_ + k_nr_) of fluorescent (f) and non-radiative (nr) transitions from the excited molecular state, this implies that an increasing rate of non-radiative transitions (possibly due to FRET) may shorten the fluorescence lifetime of the LysoTracker Green in the presence of the IOH-NPs. Therefore, in addition to direct optical excitation, IOH-NPs are probably excited via non-radiative energy transfer from the LysoTracker. Thus, total excitation of these IOH-NPs becomes more pronounced, and their fluorescence is expected to increase.

## 3. Discussion

In comparison with conventional optical imaging, multimodal fluorescence lifetime microscopy in combination with microspectrometry and laser scanning microscopy offers the following advantages:

Possible localization of IOH-NPs in the culture medium or within cells, particularly within lysosomes, on the basis of their fluorescence lifetimes, even if the size of these nanoparticles is well below the limits of conventional optical resolution. Fluorescence lifetime differs due to different contributions of non-radiative transitions to the total transition rate from the excited state of a molecule in different physicochemical environments.

Information on cellular uptake of IOH-NPs. A shortening of their fluorescence lifetime indicates that an uptake from the culture medium to the cells with a possible localization in acid compartments such as endolysosomal vesicles might occur between 2 h and 24 h after incubation, well in line with our previous findings [2,5]. However, some minor, but statistically insignificant, decreases in the fluorescence lifetimes of IOH-NPs in the culture medium (without cells) within 24 h were superposing the shortening of the fluorescence lifetime upon cellular uptake. Reference experiments of the IOH-NPs in buffer solution indicate that a general shortening of their fluorescence lifetime may occur in acid environment.

Information on additional mechanisms, e.g., FRET from organelle-specific dyes (here: LysoTracker Green) to the IOH-NPs, which may improve the optical excitation, and therefore the fluorescence intensity, of these nanoparticles. FRET from the LysoTracker Green to the nanoparticles—typically occurring within a distance around 10 nm [10]—is again strong evidence for the lysosomal location of the IOH-NPs and can be used in combination with further uptake studies of nanoparticles into cancer cells, e.g., using super-resolution microscopy (see below). However, competing mechanisms to FRET, e.g., aggregation or diverse quenching or pH effects, which might cause some shortening of fluorescence lifetimes, too, should further be considered.

So far, all experiments were performed with H8N8 murine breast cancer cells (and in some preliminary studies also with 3T3 murine fibroblasts). Application of our methods to clinically relevant human cell lines (see, e.g., Refs. [2,3,4]) will certainly strengthen the potential of these methods in the future.

All information can be stored as spectral signatures, fluorescence lifetimes, or transition rates and can be used, e.g., in high-content screening of cell cultures [16,17,18]. In addition, fluorescence lifetime images can be recorded; however, the size of individual nanoparticles is often smaller than the optical or electronic image resolution, so that images reflect only average values over a certain area of a cell. Time-correlated single-photon counting (TCSPC) imaging using a laser scanning microscope together with single-photon counting electronics [10] may offer a higher resolution of fluorescence lifetime images than an image intensifying camera system, since the point-spread function of a laser beam is often smaller than the pixel size of an image intensifying camera system.

In principle, fluorescence lifetime measurements can be combined with super-resolution microscopy, e.g., Structured Illumination Microscopy (SIM) [19,20,21], Single-Molecule Localization Microscopy (SMLM) [22,23,24], or Minflux Microscopy [25,26]. While SIM allows for a (lateral) resolution of around 100 nm, SMLM or Minflux techniques permit a resolution of only a few nanometers and, similar to CLEM, can make nanoparticles visible. However, these techniques are either rather elaborate or require high light exposure and long measuring times with possible phototoxic damage of the samples (for an overview see [27]). Super-resolution in the axial direction is attained by Total Internal Reflection (TIR) Microscopy [28], permitting excitation of the samples within an evanescent electromagnetic field in close vicinity to a substrate. Typical penetration depths of the evanescent wave are around 100 nm, but variation in the angle of light incidence (Variable-Angle Total Internal Reflection Fluorescence Microscopy, VA-TIRFM) permits a resolution below 10 nm within or close to a cell membrane [29,30]. Using TIRFM, cellular uptake of nanoparticles can thus be visualized (even if single particles are not really resolved), but measurements are restricted to the ventral side of the cells and exclude their dorsal side from detection. Nevertheless, a multimodal combination of fluorescence lifetime microscopy and VA-TIRFM may contribute to further studies of the uptake and location of IOH-NPs within cancer cells.

Finally, we should emphasize that not only in super-resolution microscopy, but also in conventional microscopy, microspectrometry, or FLIM, photophysical or phototoxic effects may occur with some impact on cell viability, fluorescence intensity, spectral properties, or fluorescence lifetimes. In accordance with our previous work with various fluorescent dyes or fluorescent proteins, we commonly used an irradiance of around 100 mW/cm^2^ and a total light exposure below 10 J/cm^2^, where we did not detect any phototoxic damage [27]. Even after a short-term increase in the irradiance up to a factor of 10, neither the fluorescence intensity, the emission spectra, nor the fluorescence lifetime changed; thus, we suppose that photophysical effects did not play a major role in our investigations.

## 4. Materials and Methods

### 4.1. Cell Culture of H8N8 Cells

Murine H8N8 cells, a basal-like breast cancer (BLBC) cell line with tumor stem cell characteristics derived from bi-transgenic WAP-T/WAP-mutp53 tumors [31], were cultured in Dulbecco’s Modified Eagle Medium (DMEM) with high glucose (4.5 g/L d-glucose; Carl Roth GmbH + Co. KG, Karlsruhe, Germany, 9005.1), supplemented with 10% fetal calf serum (FCS; Merck KGaA, Darmstadt, Germany, S0615). Cells were kept at 37 °C in a humidified incubator with 5% CO_2_.

### 4.2. IOH-NPs

[ZrO]^2+^[(CMP)_0.99_(DUT647)_0.01_]^2−^ IOH-NPs were prepared by dissolving 36.7 mg (0.1 mmol) of cytidine monophosphate sodium salt (Na_2_(CMP), Sigma Aldrich, Taufkirchen, Germany) in 50 mL of demineralized water. For fluorescence labeling, 25 nmol of DUT647 (Dyomics, Jena, Germany) was added to the [CMP]^2−^ solution. Thereafter, 0.5 mL of an aqueous solution containing 29.3 mg (0.09 mmol) of ZrOCl_2_·8H_2_O (99.9%, Sigma Aldrich, Taufkirchen, Germany) was injected, which resulted in an instantaneous nucleation of IOH-NPs. After 2 min of intense stirring, the IOH-NPs were separated via centrifugation (25,000 rpm, 15 min) and re-dispersed in H_2_O. Further details of synthesis and characterization are described in [3,5].

For the following experiments, we used IOH-NPs with a diameter of 50–60 nm at a concentration of 10 µg/mL. For this purpose, an aliquot of the IOH-NP stock suspension in demineralized water was thoroughly re-suspended and subsequently diluted in complete cell culture medium. A total of 2 mL of this working solution was added to each dish and incubated for time periods of 2 or 24 h at 37 °C and 5% CO_2_. To prevent IOH-NPs from agglomeration, they were stored at 4 °C on a shaker, protected from light.

For reference experiments, IOH-NPs were diluted in the cell culture medium or in a technical buffer solution of pH 4.0 or 7.0 (Mettler Toledo GmbH, Greifensee, Switzerland) at the same concentration as for the cell experiments. Measurements in the cell culture medium were repeated 24 h after incubation.

### 4.3. Confocal Imaging

For C-LSM and FLIM (s. below), ibidi µ-dishes (ibidi 80136, Ibidi GmbH, Gräfelfing, Germany) were used. Part of the dishes was equipped with a grid to identify individual cells. Each dish was seeded with approximately 60,000 tumor cells, and after 24 h, IOH-NPs were added for either 2 or 24 h. To visualize the interaction between IOH-NPs and lysosomal vesicles, part of the cells was co-incubated with LysoTracker Green DND-26 (Thermo Fisher Scientific, Freiburg i.Br., Germany, L7526) for 30 min immediately after incubation with the nanoparticles.

Cells were imaged in a confocal microscope (LSM 700, Carl Zeiss GmbH, Jena, Germany) using the excitation wavelengths of 555 nm (LysoTracker Green and IOH-NPs) or 639 nm (IOH-NPs). To choose cells of interest, a 10× objective lens was first used to identify the grid pattern to find the same cells again in the fluorescence lifetime microscope. Afterwards, selected cells were imaged with a 63× oil immersion objective lens at high resolution, as described in [5].

### 4.4. Fluorescence Lifetime and Microspectrometry Experiments

FLIM and microspectrometry experiments were performed in combination with confocal microscopy using cells of the same slides after incubation with IOH-NPs (2 or 24 h) or co-incubation with IOH-NPs (2 or 24 h) and LysoTracker Green DND-26 for 30 min. Cells incubated only with LysoTracker Green, as well as non-incubated cells, were used as controls.

For the acquisition of fluorescence spectra and decay kinetics of 5–10 individual cells in each case, a super-continuum fiber laser operated at (470 ± 20) nm, or at (550 ± 20) nm or at (610 ± 10) nm with 5 ps pulse duration and 78 MHz repetition rate (NKT Photonics, Birkeröd, Denmark) was coupled to an upright microscope (Axioplan 1, Carl Zeiss Microimaging GmbH, Jena, Germany) via a single mode glass fiber. The microscope was equipped with a 63×/0.90 water immersion objective lens, which was dipped into the culture medium. While in the first case (470 nm), LysoTracker Green was excited exclusively, the IOH-NPs were excited at both 550 nm and 610 nm. A long pass filter for 520 nm was used for fluorescence measurements at an excitation wavelength of 470 nm, a long pass filter for 590 nm at an excitation wavelength of 550 nm, and a long pass filter for 645 nm at an excitation wavelength of 610 nm. For acquisition of the fluorescence spectra at a resolution Δλ ≤ 10 nm, a custom-made polychromator was placed on top of the microscope and combined with an image intensifying system (IMD D4562, Hamamatsu Photonics, Ichino-Cho, Japan). To measure the fluorescence decay kinetics, we used an image intensifying camera system (Picostar HR12 image intensifier coupled to a cooled ICCD camera; LaVision, Göttingen, Germany). This system allowed us to measure the fluorescence decay curves in steps of 200 ps over a time axis of 10 ns and to fit these curves using multiple exponential components. For the present experiments, we used a tri-exponential curve fitting algorithm according to I_F_ (t) = A_0_ + A_1_ e^−t/τ1^ + A_2_ e^−t/τ2^ + A_3_ e^−t/τ3^ with the fluorescence intensity I_F_ (t); the fluorescence lifetimes τ_1_, τ_2_, and τ_3_; and the corresponding amplitudes A_1_, A_2_, and A_3_. Median values ± median absolute deviations (MADs) or mean values ± standard deviations were determined for a larger number of experiments as specified in Section 2.1 and Section 2.3.

**Figure 5:**
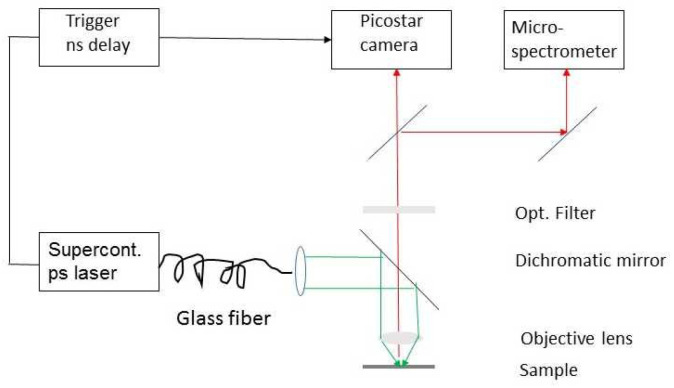
Experimental setup for combined fluorescence lifetime microscopy and microspectrometry (green: excitation beam; red: fluorescence).

The detection system also allowed us to measure fluorescence lifetime images with the “effective fluorescence lifetime” τ_eff_, calculated from the intensities I_1_ and I_2_ that were measured in two time gates of identical width and a time shift Δt between one another according to τ_eff_ = Δt/ln (I_1_/I_2_). τ_eff_ is the decay time, which would result from mono-exponential curve fitting. Due to the resolution of the multichannel plate of the image intensifier, the spatial resolution of images was limited to about 0.35 µm when using the 63× magnifying lens. In some cases, gridded ibidi µ-dishes (see above) were used to measure C-LSM and FLIM images of the same cells and thus to select cells with a sufficiently large number of IOH-NPs in the confocal microscope and to use these cells for microspectrometry and fluorescence lifetime experiments. The experimental setup for fluorescence lifetime microscopy and microspectrometry is shown in Figure 5.

## 5. Conclusions

In view of nanoparticle-based strategies to treat cancer, multimodal microscopy, including fluorescence lifetime microscopy, microspectrometry, and confocal laser scanning microscopy, can contribute to the study of cellular, e.g., lysosomal uptake of IOH-NPs, and optimization of fluorescence excitation. Optical microscopy is thus extended to 5 dimensions, including three coordinates, wavelength, and picosecond decay times.

## Figures and Tables

**Table 1:** Summary of the findings concerning IOH-NPs.

Section	Sample	Fluorescence Decay Kinetics	Fluorescence Spectra	LSM Images
2.1	H8N8	3 exponential components (Figure 3)	Autofluorescence + IOH-NPs (Figure 2)	Ref. [5]
	2 h	τ_2_ = (1.70 ± 0.10) ns (λ_ex_ = 550/610 nm)	IOH-NP band at 670/680 nm	I_555 nm_ ≤ I_639 nm_
	24 h	τ_2_ = (1.40 ± 0.24) ns (λ_ex_ = 550/610 nm) Hypothesis: Shortening after cellular/lysosomal uptake	IOH-NP band increasing	I_555 nm_ ≤ I_639 nm_ (Figure 4)
	Medium 0 h	τ_2_ = (1.68 ± 0.08) ns (λ_ex_ = 550/610 nm)	IOH-NP band at 670/680 nm	
	24 h	τ_2_ = (1.47 ± 0.14) ns (λ_ex_ = 550/610 nm) Hypothesis: Minor intrinsic change in fluorescence lifetime	IOH-NP band at 670/680 nm	
	Buffer pH 7.0	τ_2_ = (1.80 ± 0.29) ns (median) τ_2_ = (2.667 ± 1.517) ns (mean value)	IOH-NP band at 670/680 nm	
	pH 4.0	τ_2_ = (1.81 ± 0.26) ns (median) τ_2_ = (1.885 ± 0.466) ns (mean value) Mean values indicate shortening of τ_2_ with decreasing pH.	IOH-NP band at 670/680 nm; No spectral shift with pH	
2.2	H8N8	τ_eff_ = 2.5–3.5 ns all over the cells (FLIM); (λ_ex_ = 610 nm) (Supplementary Figure S1)		
2.3	LysoTr.	τ_1_ = 0.5–0.7 ns τ_2_ = (4.36 ± 0.20) ns (λ_ex_ = 470 nm)	Broad spectral band with maximum at 530–535 nm and long-wave tail up to 730 nm	
	LysoTr. + IOH-NPs	τ_1_ = 0.5–0.7 ns τ_2_ = (4.05 ± 0.30) ns (λ_ex_ = 470 nm), Shortening explained by FRET LysoTracker ⟶ IOH-NPs		I_555 nm_ ≥ I_639 nm_ indicating additional excitation via FRET (Figure 4)

## Data Availability

The original contributions presented in this study are included in the article/Supplementary Material. Further inquiries can be directed to the corresponding author.

## Supplementary Materials

The following supporting information can be downloaded at https://www.mdpi.com/article/10.3390/ijms27031339/s1.

## Abbreviations

The following abbreviations are used in this manuscript:

IOH-NPs	Inorganic–organic hybrid nanoparticles
CLEM	Correlative light and electron microscopy
FRET	Förster resonance energy transfer
FLIM	Fluorescence lifetime imaging (microscopy)
C-LSM	Confocal laser scanning microscopy
TIR(FM)	Total internal reflection (fluorescence microscopy)
MADs	Median absolute deviations
