# Supplementary material for: Monitoring Nanoparticle Interaction with Murine Breast Cancer Cells Using Multimodal Fluorescence Lifetime Microscopy
Source: Int J Mol Sci. 2026 Jan 29;27(3):1339. doi: 10.3390/ijms27031339 (PMC12898725; doi:10.3390/ijms27031339)
Supplement: Supplementary file 1 [file ijms-27-01339-s001.zip › Nanoparticles_Lifetime_ijms_Supplementary Figure_1.pdf]

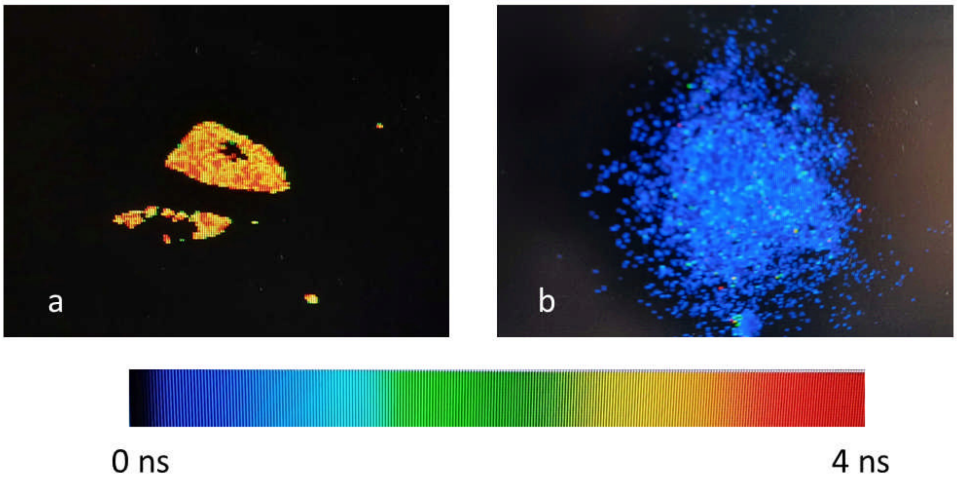

Supplementary Figure S1. FLIM of 2 H8N8 cells incubated for 24 h with IOH-NPs (a) and FLIM of about 5 non-incubated H8N8 control cells (without discernable structure) (b); excitation wavelength: (610 ± 10) nm; detection range: ≥ 645 nm; pixel resolution: 0.35 μm; image size: 240 μm × 160 μm.
