# Supplementary material for: Monitoring Nanoparticle Interaction with Murine Breast Cancer Cells Using Multimodal Fluorescence Lifetime Microscopy
Source: Int J Mol Sci. 2026 Jan 29;27(3):1339. doi: 10.3390/ijms27031339 (PMC12898725; doi:10.3390/ijms27031339)
Supplement: Supplementary file 1 [file ijms-27-01339-s001.zip › Nanoparticles_Lifetime_Values.pdf]

**Fl. Decay Time τ_2 in ns (2h)**	**Dev. From Median in ns**	**Fl. Decay Time τ_2 in ns (24h)**	**Dev. From Median in ns**
4.16	2.40	2.64 (*)	1.24
2.22	0.52	2.60 (*)	1.20
1.86	0.33	2.18	0.78
1.86	0.29	2.01 (*)	0.61
1.86	0.25	2.01 (*)	0.61
1.86	0.25	1.97 (*)	0.57
1.82	0.25	1.88 (*)	0.48
1.80	0.19	1.76	0.46
1.79	0.16	1.55	0.41
1.78	0.16	1.52	0.40
1.77	0.16	1.50	0.36
1.75	0.16	1.42	0.34
1.74	0.14	**1.41**	**0.28**
1.74	0.12	**1.40 (*)**	**0.20**
1.74	0.12	1.38	0.19
1.72	0.11	1.32	0.15
**1.70**	**0.10**	1.32	0.15
1.68	0.09	1.31 (*)	0.13
1.68	0.09	1.25	0.10
1.66	0.08	1.21	0.09
1.64	0.08	1.20 (*)	0.08
1.64	0.07	1.12 (*)	0.08
1.62	0.06	1.06	0.02
1.61	0.06	1.00 (*)	0.01
1.59	0.05	0.99	0.01
1.58	0.04	0.94	0.00
1.56	0.04		
1.51	0.04	(*) = 610 nm Exc.	
1.45	0.04	Other = 55 0 nm Exc.	
1.45	0.02		
1.45	0.02		
1.41	0.02		
1.37	0		
Median: 1.70	MAD: 0.10	Median: 1.40	MAD: 0.24

Incubation with LysoTracker / FRET experiments

**Fl. Decay Time τ_2 in ns without IOH-NPs**	**Deviation from Median (ns)**	**Fl. Decay Time τ_2 in ns with IOH-NPs**	**Deviation from Median (ns)**
4.89	0.53	5.24	1.25
4.67	0.40	4.54	1.19
4.65	0.36	4.44	0.81
4.62	0.29	4.36	0.80
4.40	0.26	4.29	0.67
**4.36**	**0.20**	4.24	0.49
4.35	0.16	4.12	0.39
4.34	0.04	4.12	0.37
4.20	0.02	4.12	0.31
4.16	0.01	3.98	0.29
3.96	0.00	3.89	0.22
		3.87	0.19
		3.83	0.18
		3.68	0.16
		3.38	0.07
		3.25	0.07
		3.24	0.07
		2.90	0.07
Median: 4.36	MAD: 020	Median: 4.05	MAD: 0.30

IOH-NPs in buffer solution:

**pH 7 Decay Time τ_2 in ns**	**Deviation from Median (ns)**	**pH 4 Decay Time τ_2 in ns**	**Deviation from Median (ns)**
6.26	4.46	3.16	1.35
5.12	3.32	2.50	0.69
5.08	3.28	2.26	0.53
3.40	1.60	2.05	0.45
2.84	1.04	2.03	0.31
2.64	0.84	1.99	0.30
2.34	0.54	1.94	0.28
1.84	0.30	1.86	0.28
1.76	0.28	1.77	0.24
1.73	0.20	1.66	0.22
1.72	0.19	1.59	0.22
1.72	0.08	1.53	0.18
1.61	0.08	1.53	0.15
1.60	0.07	1.51	0.13
1.52	0.04	1.50	0.05
1.50	0.04	1.28	0.04
Median: 1.80	MAD: 0.29	Median: 1.81	MAD: 0.26
Mean: 2.667	Stand. Dev.: 1.517	Mean: 1.885	Stand. Dev.: 0.466

IOH-NPs in culture medium:

**Fl. Decay Time τ_2 in ns (0 h)**	**Dev. From Median in ns**	**Fl. Decay Time τ_2 in ns (24 h)**	**Dev. From Median in ns**
2.64	0.96	2.02	0.55
2.55	0.87	1.90	0.43
1.80	0.22	1.89	0.42
1.76	0.19	1.82	0.35
1.75	0.16	1.62	0.15
1.70	0.12	1.58	0.15
1.66	0.08	1.48	0.15
1.66	0.08	1.46	0.13
1.61	0.07	1.45	0.09
1.61	0.07	1.38	0.09
1.52	0.07	1.38	0.09
1.49	0.02	1.34	0.02
1.46	0.02	1.32	0.01
		1.32	0.01
Median: 1.68	MAD: 0.08	Median:1.47	MAD: 0.14
